# Trisomy of Human Chromosome 21 Orthologs Mapping to Mouse Chromosome 10 Cause Age and Sex-Specific Learning Differences: Relevance to Down Syndrome

**DOI:** 10.3390/genes12111697

**Published:** 2021-10-26

**Authors:** Ross Minter, Katheleen J. Gardiner

**Affiliations:** 1 Linda Crnic Institute for Down Syndrome, University of Colorado Anschutz Medical Campus, Aurora, CO 80045, USA; rmbminter@gmail.com; 2 Department of Pediatrics, University of Colorado Anschutz Medical Campus, Aurora, CO 80045, USA

**Keywords:** Dp10(1)Yey, intellectual disability, prefrontal cortex, hippocampus, striatum, puzzle box, double-H maze, prepulse inhibition, female, aging

## Abstract

Down syndrome (DS), trisomy of human chromosome 21 (Hsa21), is the most common genetic cause of intellectual disability. The Dp10(1)Yey (Dp10) is a mouse model of DS that is trisomic for orthologs of 25% of the Hsa21 protein-coding genes, the entirety of the Hsa21 syntenic region on mouse chromosome 10. Trisomic genes include several involved in brain development and function, two that modify and regulate the activities of sex hormones, and two that produce sex-specific phenotypes as null mutants. These last four are the only Hsa21 genes with known sexually dimorphic properties. Relatively little is known about the potential contributions to the DS phenotype of segmental trisomy of Mmu10 orthologs. Here, we have tested separate cohorts of female and male Dp10 mice, at 3 and 9 months of age, in an open field elevated zero maze, rotarod, and balance beam, plus the learning and memory tasks, spontaneous alternation, puzzle box, double-H maze, context fear conditioning, and acoustic startle/prepulse inhibition, that depend upon the function of the prefrontal cortex, striatum, hippocampus, and cerebellum. We show that there are age and sex-specific differences in strengths and weaknesses, suggesting that genes within the telomere proximal region of Hsa21 influence the DS phenotype.

## 1. Introduction

Human chromosome 21 (Hsa21) encodes ~165 classical protein-coding genes, ~50 members of the Keratin Associated Protein family (KRTAP), and several hundred other transcribed sequences that may encode functional RNAs, short open reading frames, or merely transcriptional noise [1]. Some subset of these genes, when present in three copies, contributes to the phenotypic features of Down syndrome (DS), trisomy of Hsa21. The full phenotype of DS is complex, highly variable in penetrance and severity, and can affect many organs and organ systems [2,3]. Common to all with DS is some level of intellectual disability (ID) which most often is moderate to severe [4,5,6]. Impairments in executive function, some spatial navigation strategies, and language skills are well documented in DS [7,8,9,10,11,12], implicating the prefrontal cortex, hippocampus, and cerebellum, respectively [7,13,14,15,16], although there are recent reports of enhanced spatial learning in strategies implicating the striatum [17,18]. The DS brain is characterized by decreased hippocampal and cerebellar volumes, decreased neuronal densities, and altered dendritic spine morphologies [10,19]. Other neurological features seen in DS include the increased incidence of seizures and autism, and the development of the neuropathology of Alzheimer’s Disease (AD) early in life, and early onset AD-like dementia [20,21]. The worldwide incidence of DS is one in ~1000–2000 live births, and the average lifespan now exceeds 60 years in many countries [22,23,24,25,26,27]. Thus, DS represents a significant societal challenge. Preventing or controlling ID and other neurological symptoms could be possible with appropriate pharmacotherapies. While rationale development of such therapies might be facilitated by understanding the functional features of individual Hsa21 genes, achieving such a goal is complicated by the existence of direct and indirect interactions among Hsa21 protein-coding genes [28]. Examining the practical effects of trisomy, therefore, requires the study of segmental trisomies—model systems that overexpress many, if not all, Hsa21 genes or orthologs. 

Orthologs of Hsa21 protein-coding genes map to segments of three mouse chromosomes: Mmu 16, 17, and 10. This distribution has meant that complete trisomy mouse models have been challenging to construct and slow in coming [29,30,31,32,33]. Most research with segmental trisomy mice has been directed, for historical reasons, to the ~65% of Hsa21 orthologs mapping to the Mmu16 segment, and mice trisomic for all or part of this region present with many features relevant to DS, including learning and memory (LM) impairments and brain structure abnormalities [34,35,36,37,38]. However, the Mmu10 orthologous region contains ~25% of Hsa21 protein-coding orthologs [1], and many of these 39 genes display interesting characteristics when mutated or altered in expression level. For example, overexpression of the calcium-binding protein, S100B, causes LM deficits [39,40]; in cell systems, endostatin, a proteolytic product of collagen 18A1, inhibits neurite outgrowth [41]; and mutations in the protease inhibitor, cystatin B (CSTB), cause epilepsy and its deletion prevents the development of cognitive deficits in a model of AD [42,43]. Other Mmu10 orthologs have sex-specific consequences, either normally or when mutated. These include the protein arginine methyltransferase, PRMT2, and the small ubiquitin-like modifier protein, SUMO3, which modify and regulate the activities of the estrogen, progesterone, and androgen hormone receptors [44,45,46,47,48], and null mutations of the pre-mRNA deaminase, ADARB2, and the transient receptor channel, TRPM2, that produce phenotypes in male but not female mice [49,50,51]. Thus, based on gene functions, trisomy of the Mmu10 orthologous segment would be predicted to display behavior and/or LM abnormalities and, potentially, sex differences in phenotypic features. 

The segmental trisomy mouse, Dp(10)1Yey (abbreviated Dp10), is trisomic for the entire Mmu10 segment orthologous to Hsa21 [31]. Significant perturbations in protein expression in the brains of these mice have been reported to differentially affect normal sex differences in protein levels with brain region-specific patterns [52,53]. In 8-month-old naive Dp10 mice, abnormal protein levels in the cortex, hippocampus, and cerebellum were shown to include multiple components of MTOR, MAPK and apoptosis pathways, and glutamate receptor subunits. Importantly, these trisomy abnormalities predominantly affected the hippocampus in male Dp10 and cerebellum in female Dp10. These sex differences are important for two reasons. First, each of the pathways and proteins assayed was chosen because of their well-established roles in normal LM processes, ID, and/or AD [28,52]. That levels can show sex-specific perturbations suggests potential sex differences in phenotypic consequences of trisomy. Second, consistent with, and potentially relevant to, sex-specific protein expression differences, sex differences in cognitive profiles of people with DS have been reported. Females with DS outperformed males on a subset of coding and visuospatial processing tasks [54], visual associative memory, verbal episodic memory and language comprehension, and executive functions involving working memory, planning, and mental flexibility [55]. They also developed earlier and better speech production abilities and were more likely to show mild ID than males [56,57]. Given that the only Hsa21 genes with known sex-specific functions map to Mmu10, it is particularly important to explore potential sex-specific phenotypic features of the Dp10 mice.

In spite of the functional properties of genes trisomic in the Dp10 mice and perturbations in protein expression, initial studies of male Dp10 mice at 2–4 months of age revealed no deficits in the Morris Water Maze (MWM) or context fear conditioning (CFC) [31]. More recently, however, 3-, 6- and 9-month-old Dp10 mice were shown to be impaired in a spatial working memory spontaneous alternation task [58]. Currently, LM studies of Dp10 mice have examined only male mice and used tasks requiring a functional hippocampus. To expand the characterization of these mice, we have examined female and male mice at ~3 months and ~9 months of age, for the performance of several behavioral features and in LM tasks that require medial prefrontal cortex, striatum, and cerebellum, as well as the hippocampus. We show that performance features are influenced by sex, age, and brain region.

## 2. Materials and Methods

### 2.1. Mice

All mice were bred at the University of Colorado Anschutz Medical Campus from an original breeding pair gifted from Y. E. Yu (Roswell Park, NY, USA). Colonies were maintained in ventilated cages in a room with HEPA filtered air and a 14:10 h light:dark cycle, and fed a 6% fat diet and acidified (pH 2.5–3.0) water ad libitum. Trisomic Dp10 males were crossed with wild-type C57BL/6J females. Sex-matched littermates were housed together (leading to groups of 1–5 per cage), keeping non-breeding females in diestrus. Mice were genotyped using a standard PCR protocol (see [53] for additional details). A total of 111 mice, obtained from 26 litters and deriving from 13 dams and 10 sires, were used. Mice were analyzed in separate cohorts of two ages, ~3 months and ~9 months; numbers in each cohort are provided in Table 1 and are the *n* values for each experiment except fear conditioning (see below). Power analysis indicates that these numbers are adequate, based on results reported in the literature for behavioral testing and our experience with DS lines, for detection of a 15% difference, with *p* < 0.05 and 15% standard deviation, with a power of 0.909. Information for each mouse, including littermates, birth and sacrifice dates, and weight, are provided in Supplementary Table S1.

### 2.2. Behavioral and Cognitive Testing

**Figure 1:**
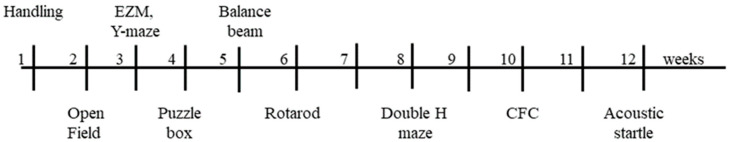
Timeline. Order and time frame of tasks. Numbers indicate the week each task was carried out. Weeks with no task, mice were allowed to rest to reduce stress. EZM, elevated zero maze. CFC, context fear conditioning.

Behavioral and cognitive assays spanned ~3 months. All mice were handled two minutes a day for 2 days prior to the first assay. All testing occurred between 800−1300 h to control for circadian effects, with the exception of rotarod, which was conducted 1300−1700 h. Each day prior to the start of testing, mice were acclimated to the experimental room in their home cages for 30 min. The experimenter was blind to genotype while testing the mice. All mice underwent behavioral and cognition evaluations in the same order and over a similar time frame (Figure 1); mice were rested for two weeks to decrease accumulated stress that might affect learning, before and after the more stressful tests of the double-H maze, fear conditioning, and acoustic startle. In all behavioral and cognitive assays, after a mouse was removed from the apparatus, the equipment was cleaned, and 70% ethanol was applied.

All procedures were approved by the University of Colorado Institutional Animal Care and Use Committee and performed in accordance with the National Institute of Health guidelines for the care and use of animals in research.

#### 2.2.1. Behavioral Tasks

##### Open Field

Open field assays locomotion, general exploratory behavior, and anxiety. The open field arena was 44 × 44 cm, with 25 cm high walls. Under bright lighting conditions, mice were allowed to explore the open field for one 10-min trial. Total distance (cm) traveled and time spent in the outer and center zones was monitored using the video tracking system Ethovision (Noldus, Leesburg, VA, USA).

##### Elevated Zero Maze

The elevated zero maze [59], similar to the plus maze [60], measures anxiety; however, the former lacks the ambiguous center area of the latter [61]. The elevated zero maze is comprised of a circular pathway (50 cm in diameter and 5 cm wide) 50 cm above the ground. The runway is divided into four 90-degree quadrants; two opposing quadrants have walls 30 cm high, while the other two have no walls. Each mouse was introduced to the maze in the same walled quadrant and allowed to explore for 10 min. The number of zone transitions, distance moved, and percentage of time in the open and closed zones were monitored using the video tracking system Ethovision (Noldus, Leesburg, VA, USA).

##### Rotarod

The rotarod [62,63] is a measure of sensorimotor function, balance, and equilibrium. The cylinder (3 cm diameter), which rotates, has five lanes separated by dividers (Med Associates, Georgia, VT, USA). Fifteen cm below the cylinder are photo beams that record the time of any falls. Each mouse was placed forward-facing (opposite of the rotation direction) on the stationary rotarod. Trials began at 3 RPM and gradually increased to 30 RPM over the course of 5 min. Each mouse received 3 trials with a 10-min delay between each. Latency to fall was measured using Med Associates software (Fairfax, VT, USA).

##### Balance Beam

Balance Beam, modeled after [64], is a measure of motor coordination. Mice underwent an acclimation day followed by a testing day. During acclimation, mice were placed in the dark goal box, with bedding, 50 cm above the ground, for 3 min. Mice then completed 4 training trials. Mice were placed on a 12 mm square metal beam at increasing distances from the goal box (10, 20, 40, 80 cm). If they froze or headed in the wrong direction, mice were gently encouraged to proceed. Once in the goal box, mice were allowed to rest for 30 s. On the testing day, starting with the largest beam and at 80 cm from the goal box, mice were tested sequentially twice each on three beams of decreasing width (28, 12, 5 cm). Time to reach the goalbox was measured, and the two trials for each beam width were averaged. If a mouse fell off the beam, it was placed back at the start, and the timer was reset. If 3 consecutive falls occurred on the same beam size, testing was stopped. Once in the goal box, mice were allowed to rest for 30 s.

#### 2.2.2. Learning/Memory Tasks

##### Spontaneous Alternation

Spontaneous alternation [65,66] is a measure of working spatial memory. The testing occurs in a Y-shaped maze (45 cm, each arm) with arms at 120-degree angles. A spontaneous alternation is defined as visiting each of the three arms without revisiting the same arm until all other arms are visited first. Mice were placed in the center of the maze, and arm entries were measured for 8 min or until the mouse completed 22 arm entries. Arm choices were observed using the video tracking system Ethovision (Noldus, Leesburg, VA, USA).

##### Puzzle Box

Puzzle box [67] is a measure medial prefrontal cortex (mPFC) and executive function. The box is comprised of two compartments: a bright, open start zone (58 cm long, 28 cm wide) and a smaller dark, enclosed goal zone (15 cm long, 28 cm wide). Typically, mice will move from the start zone to the goal zone to escape the brightly lit open space. Mice underwent three trials per day for three consecutive days (day 1: T1–T3, day 2: T4–T6, day 3: T7–T9). During T1, there was no barrier to entering the goal zone through a narrow open doorway (4 cm × 10 cm). During T2–T9, that doorway was blocked, and the mouse was required to find and use an underpass to reach the goal zone. During T2–T4, the underpass was open. During T5–T7, the underpass was filled with sawdust, and the mouse was required to dig through the underpass. During T8 and T9, the underpass was further covered with sawdust plus a 4 × 4 cm piece of cardboard, and the mouse was required to use teeth to remove this before digging through the underpass. Each trial ended when the mouse reached the goal zone or after 300 s; the inter-trial interval was 20 min. Latency to reach the goal zone was measured using the video tracking system Ethovision (Noldus, Leesburg, VA, USA).

##### Double-H Maze

The double-H maze [68] is a water maze that can be used, depending on the protocol, to assay egocentric learning to uncover deficits in striatum function. The maze is constructed of transparent plexiglass and composed of a central corridor (80 × 10 × 30 cm) bisected by three arms (80 × 10 × 30 cm), forming, as the name implies, an H shape [68]. An escape platform is placed in one arm. The maze is in a room with well-contrasted cues on the walls. The water was made opaque with non-toxic paint. Mice were familiarized with swimming in water one day prior to testing. Mice of each sex/genotype cohort were divided into two groups which differed in the patterns of turns required to locate the escape platform, right-left (RL) or left-right (LR). At the start of each trial, the mouse was placed in the N or S arm of the maze (pseudorandom order, with each arm being used for an equal number of trials, and without three consecutive starts in the same location), with the opposing arm blocked by a guillotine door. The goal platform was placed according to which group (RL or LR) the mouse was in. Latency to reach the goal platform, the number of errors, and working memory errors were recorded with a video tracking system. A trial ended when the mouse reached the goal platform or 60 s had passed; the mouse was allowed to spend 15 s on the platform. Each mouse received 4 trials per day, with 3 min between trials, for 4 days.

##### Context Fear Conditioning

Context fear conditioning (CFC) was performed largely as described [69,70]. Briefly, in a context-shock (CS) training session, mice were placed in a novel cage (Med Associates, St. Albans, VT, Modular Mouse Test Chamber), allowed to explore for three minutes and then given a single electric shock (2 s, 0.7 mA, constant electric current). Mice were returned to the home cage, and 60 min later, in a CS testing (CS-t) session, mice were re-exposed to the same context for 3 min without any electric shock. In both CS and CS-t sessions, the time spent “freezing” (the absence of movement except for respiration) was measured. In a shock-context (SC) training session, a second group of mice was placed in the novel cage, immediately given the electric shock (2 s), and then allowed to explore for 3 min. After 60 min in the home cage, these mice were returned to the same context for an SC testing (SC-t) session, where they were allowed to explore for 3 min in the absence of any electric shock. Time spent freezing was measured in both SC and SC-t sessions. Freezing is indicative of associative learning between the aversive experience (shock) and the context in which the shock was received; in normal control mice, freezing is significantly higher in the CS-t group.

##### Acoustic Startle and Prepulse Inhibition

Acoustic Startle [71] and prepulse inhibition (PPI) measure cerebellar learning that does not involve motor coordination. On day 1, mice were acclimated to the apparatus with two trials of 5 min in the presence of 65 dB white noise. Other researchers have allowed for more acclimation trials; however, we saw a substantial reduction and, in most cases, no fecal and urine discharge after the first acclimation. We believe this is due to the weeks of testing these mice have undergone before this task and felt that additional acclimation trials were unnecessary. On day 2, mice underwent acoustic startle testing, which consisted of a 5-min acclimation, 30 startle-only trials (a burst of 110 dB), 48 prepulse inhibition trials (prepulse + startle, startle-only, prepulse-only), followed by 9 additional startle-only trials. Prepulse + startle trials had three different prepulse intensities (74, 78, 82 dB) and two interstimulus intervals (30 and 100 ms). Startle-only trials were all 110 dB. This volume was determined using the i/o function in WT mice not part of these experimental groups; for details, see [69]. Prepulse-only trials consisted of just a prepulse at the three intensities (74, 78, 82 dB). The 48 prepulse inhibition trials were broken into six blocks. Each block included each of the six different prepulse + startle, one startle-only, and one of the three prepulse-only trials. The order within each block was randomized, and each mouse received the same order during prepulse inhibition trials. The % PPI was calculated using the following equation: prepulse + startle amplitude average/startle-only average (during prepulse inhibition trials)*100.

##### Statistical Analysis

Eight groups of mice were analyzed: female and male, control and trisomic, each at 3 and 9 months. Of the 28 possible pairwise comparisons, 12 are of logical interest to describe the effects of sex, trisomy, and age. Statistical analyses were conducted using GraphPad Prism version 8.4.2 (GraphPad, San Diego, CA, USA). Genotype, age, and sex differences were determined using statistical tests common for each cognitive assessment, including Three-way Analysis of Variance (ANOVA) to determine interactions among the three factors (sex, genotype, and age), followed by Sidak’s multiple comparison test, Repeated measures ANOVA, followed by Bonferroni correction, and non-parametric *t*-tests. Spearman correlation analysis was used to determine the relationship between weight and other parameters affecting rotarod performance.

## 3. Results

### 3.1. Behavioral Assays

#### 3.1.1. Open Field and Elevated Zero Maze

**Figure 2:**
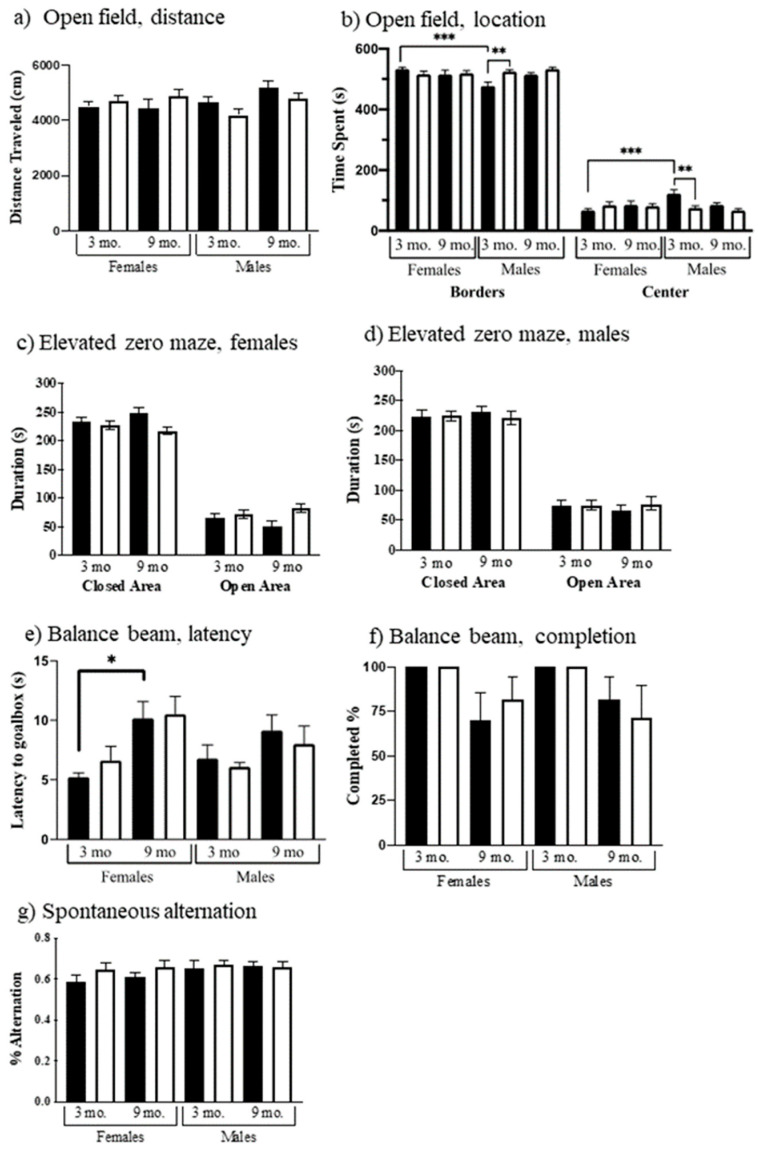
Performance in basic behaviors. Black bars, control; white bars, Dp10. Open field: (**a**) Total distance traveled, (**b**) Females and males, time at borders, and time in center. Elevated zero maze: (**c**) Females, open and closed areas; (**d**) Males, open and closed areas. Balance beam: (**e**) Time to reach goal box on narrowest beam; (**f**) % animals that completed the narrowest beam crossing. Spontaneous alternating Y-maze: (**g**) Females and males. Significant differences are indicated, *p* < 0.05, *p* < 0.01, *p* < 0.001 with *, **, and ***.

General locomotor activity, exploratory behavior in a novel environment, and anxiety can be assessed in the open field and elevated zero maze. Distance traveled was affected by the interaction of sex x genotype (F(1, 103) = 5.874, *p* = 0.0171); however, no significant differences were detected using multiple comparisons (Figure 2a). All groups showed the expected preference for the border areas, however, there are genotype (F(1, 101) = 4.116, *p* = 0.0451), age x sex (F(1, 101) = 5.348, *p* = 0.0228), and genotype x sex (F(1, 101) = 9.358, *p* = 0.0028) effects in exploratory activity/anxiety (Figure 2b). Three-month-old control males spent significantly more time in the center and less time in the borders compared to three-month-old control female mice (*t* = 4.199 *p* = 0.0007) and to three-month-old Dp10 males (*t* = 3.567 *p* = 0.0066).

In the elevated zero maze, mice again are allowed to explore a novel environment, this time in a circular apparatus with open and closed areas. Genotype showed significant effects in time spent in the open or closed areas. Dp10 mice spent more time than control in the open areas (F(1, 103) = 4.098, *p* = 0.0455). No significant differences were detected using multiple comparisons (Figure 2c,d).

#### 3.1.2. Rotarod and Balance Beam

**Figure 3:**
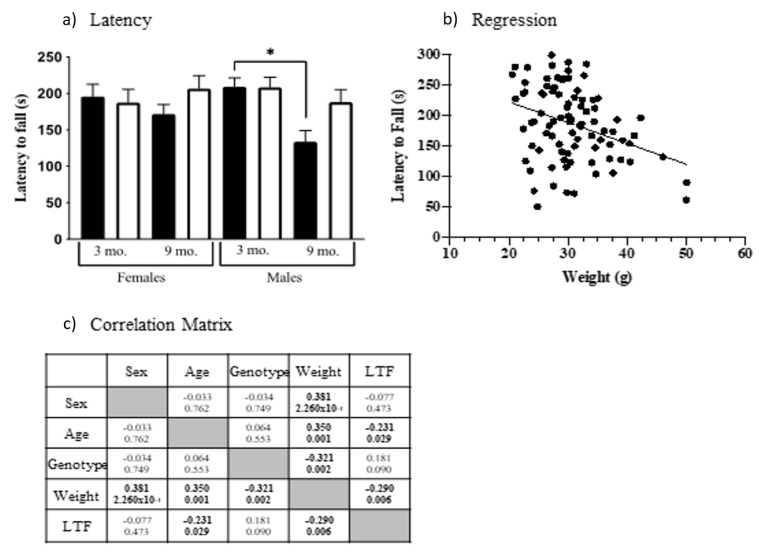
Rotarod performance. (**a**) Latency to fall. Black bars, controls; white bars, Dp10. Significant differences are indicated with *. (**b**) Regression, mouse weights vs. latency to fall. (**c**) Spearman correlation, *r*-values, and *p*-values; significant correlations are shown in bold.

One measure of coordination and balance uses the accelerating rotarod. Mice were exposed to three trials and when latencies were averaged, age F(1, 103) = 5.006, *p* = 0.0274) and the interaction of age x sex F(1, 103) = 4.122, *p* = 0.0449) and age x genotype F(1, 103) = 4.831, *p* = 0.0302) were significant. As shown in Figure 3a, 9-month-old control mice have a significantly shorter latency to fall than 3-month-old control males (*t* = 3.319, *p* = 0.0149). Male Dp10 mice do not show this impairment.

Because weight can affect physical abilities, including rotarod performance [72], we examined the relationship between latency to fall and weight, demonstrating that they are correlated (Spearman correlation analysis, Figure 3b, *r* = −0.290, *p* = 0.006). This analysis also revealed 9-month-old mice were heavier than 3-month-old mice and, interestingly, that male controls were heavier than male Dp10 (Correlation matrix, Figure 3c). Although this last is contrary to observations in people with DS, these results are consistent with the relative abilities in the rotarod of 9-month-old male controls and Dp10 mice.

Balance and coordination were also assessed as time to cross a square metal beam from a lighted box to a preferred dark box. Three beam widths were used, starting with the widest. Results for the narrowest beam are shown in Figure 2e. Latencies of 9-month-old mice were greater than those of 3-month-old mice (F(1, 63) = 16.41, *p* = 0.0001). Additionally, for 9-month-old female controls, the latency was almost twice that of 3-month-old females (*t* = 3.096, *p* = 0.0346); this difference is not significant for Dp10 females. Nine-month-old mice of both genotypes and sexes were more likely to fall off the beam (F(1, 76) = 12.75, *p* = 0.0006) (Figure 2f), with some mice failing to complete a single trial on the narrow beam.

### 3.2. Learning and Memory Assessments

#### 3.2.1. Spontaneous Alternation

As shown in Figure 2g, no sex, genotype, or age differences were observed in working spatial memory as measured using spontaneous alternation. All groups of mice showed alternation rates equal to or greater than ~60%.

#### 3.2.2. Puzzle Box

**Figure 4:**
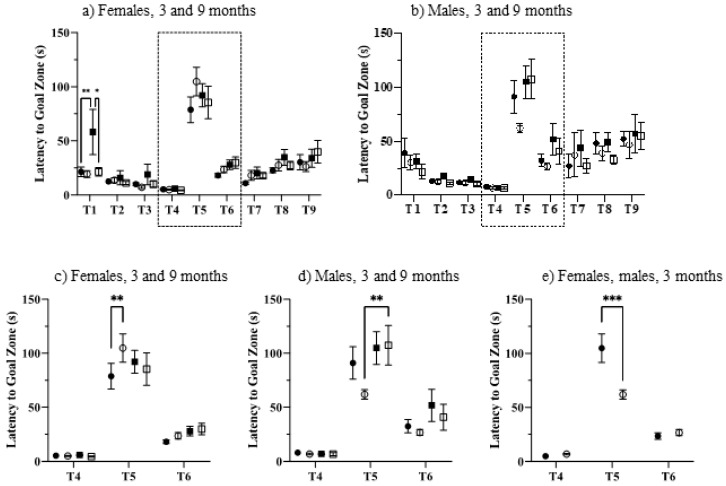
Puzzle box. (**a**–**d**), 3-month, control, filled circles; Dp10, open circles; 9-month control, filled squares; Dp10, open squares. (**c**) 3 month, Dp10 female, filled circles; Dp10 male, open circles. Dashed boxes in (**a**,**b**) indicated areas enlarged in (**c**,**d**). (**e**) 3-month Dp10, females, filled circle; 3-month Dp10, males, open circle. *, *p* ≤ 0.05; **, *p* ≤ 0.01; ***, *p* ≤ 0.001.

The puzzle box is a problem-solving task designed to assess executive function, where mice are challenged to escape from a brightly lit start box to a dark goal box [67]. Mice were exposed to 9 trials, increasing in difficulty. In Trials 1–4, there was no barrier between the start area and the goal area. In Trial 1, both sexes, genotypes, and ages escaped in ~20 s (Figure 4a,b), with the exception of 9-month-old female controls, who were initially very slow, requiring ~60 s (Figure 4a), significantly slower than both 9-month-old Dp10 females (*t* = 3.098, *p* = 0.0199) and 3-month-old control females (*t* = 3.485, *p* = 0.0053). In Trials 2–4, there were no significant differences between any groups, as all mice reached escape times of ~10 s.

Trial 5 was the first exposure to a blocked escape route; mice were required to dig their way through the underpass that was now blocked with sawdust. In this trial, all groups required ~75–110 s to escape. As shown in the enlargements in Figure 4c–e, among 3-month-old mice, sex and genotype were significant characteristics. At three months, female Dp10 were impaired compared to female controls, requiring 110 s to escape compared with only 80 s for controls (*t* = 3.525, *p* = 0.0045; Figure 4c). Among males, 9-month-old Dp10 required significantly more time than 3-month-old Dp10 (*t* = 3.575, *p* = 0.0038; Figure 4d). Lastly, 3-month-old female Dp10 were impaired compared to male Dp10, who required only 60 s (*t* = 4.211, *p* = 0.0003; Figure 4e). Differences among groups in Trial 5 were not due to physical impairments because during Trials 6 and 7, where the route remained blocked with the same sawdust barrier, all groups significantly improved their escapes times, to ~25 s for females and ~35 s for males. Notably, also in trials 6 and 7, female mice, control and Dp10, of both ages, showed little variation in escape times among individuals within the same group. In contrast, males differed considerably in within-group escape times in these trials. In both trials, this variability was significantly increased between males and females for all genotype and age comparisons (*p* < 0.0001 to 0.005; with the exception of 3-month-old Dp10 in Trial 6).

In trials 8 and 9, the increased physical difficulty of escape (cardboard added), necessitating the use of teeth as well as paws, resulted in increased escape times for all groups, with no significant differences. Males again showed significantly more variation in escape times within a group than did females, between 3-month-old controls in Trial 8 and between 3-month-old Dp10 and 9-month-old controls in Trial 9 (*p* <0.0001, 0.0009, and 0.01, respectively).

#### 3.2.3. Double-H Maze

**Figure 5:**
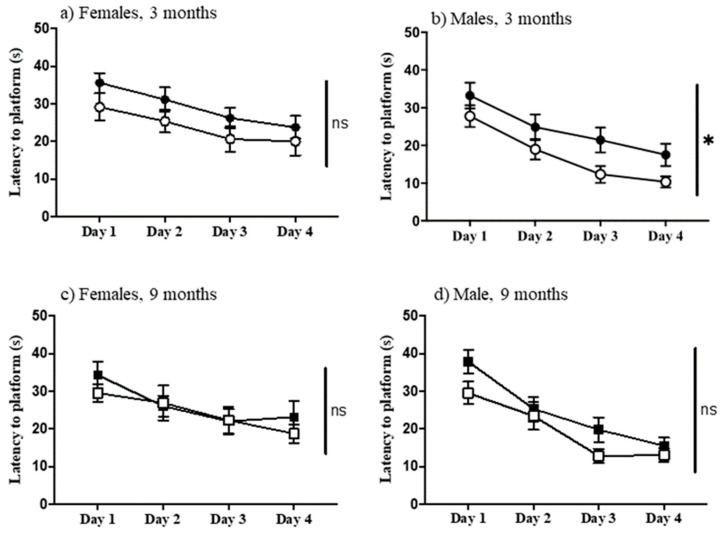
Double-H maze. Latency to reach the platform on successive days. (**a**) Females, 3-month-old; (**b**) Males, 3-month-old; (**c**) Females, 9-month-old; (**d**) Males 9-month-old. Filled circles and boxes, controls; open circles and boxes, Dp10; *, significant difference; ns, not significant.

The protocol used here for the double-H maze requires a functional striatum, i.e., to find the escape platform, mice cannot rely on extra-maze cues, but instead must learn an egocentric pattern of turns. The correct pattern of turns is successful regardless of starting point within the maze. Figure 5 shows that, unique among the age, sex, and genotype comparisons, three-month-old male Dp10 mice learned significantly better than age and sex-matched controls (F(1, 25) = 5.219, *p* = 0.031) (Figure 5b).

#### 3.2.4. Context Fear Conditioning

**Figure 6:**
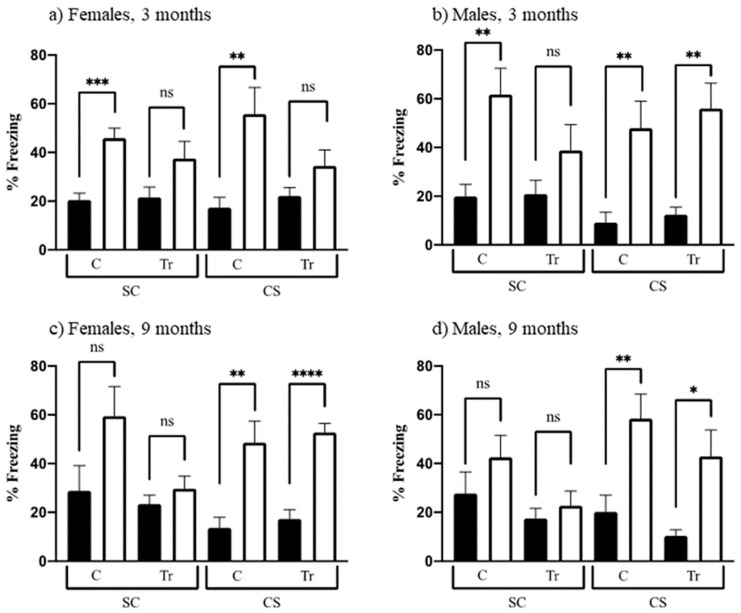
Context fear conditioning. Percent time freezing in training (black bars) and test sessions (white bars). SC, shock-context; CS context-shock. C, control; Tr, Dp10; ns, not significant. (**a**) 3-month-old females; (**b**) 3-month-old males; (**c**) 9-month-old females; (**d**) 9-month-old males. *, *p* = 0.015, **, *p* = 0.002–0.008; ***, *p* = 0.0002; ****, *p* < 0.0001+.

Learning to associate a novel context with an aversive experience requires a functioning hippocampus; here, an electric shock is used. This associative learning is demonstrated in mice by their freezing when returned to the training context. Mice were divided into two groups: the group that was allowed to explore prior to experiencing the shock (the CS group) is expected to learn and therefore freeze at relatively high levels; the group that is shocked first and then allowed to explore (the SC group) typically does not learn and subsequently exhibits a very low level of freezing. Figure 6 shows the levels of freezing, in the SC and CS training and testing sessions, for all groups. Learning can be assessed by comparing levels of freezing in CS-t to CS; alternatively, to control for the effects of the electric shock alone, levels in CS-t can be compared to those in SC-t. We have used the latter comparison previously to test several DS mouse models, including the Dp10 [73]. However, results here differed from those obtained in prior studies: for most groups, levels of freezing in CS-t are not significantly greater than those in the corresponding SC-t group, largely because, as shown in Figure 6, levels of freezing in SC-t are quite high, >35% and as high as 55–60%, for all groups except 9-month-old female and male Dp10. Indeed, for female and male 3-month-old controls, SC-t levels are significantly greater than SC levels (Figure 6a,b), suggesting that these groups unexpectedly learned to recognize the context. We have no explanation for this, although we note that the extensive experience afforded these mice during 3 months of testing may be causative.

Based on these observations, we assessed learning by comparing freezing levels in CS-t to those in CS. As shown in Figure 6a, these levels are significantly different for 3-month-old female controls but not for age-matched female Dp10, suggesting that the latter are impaired in this task. In contrast, 3-month-old male mice, controls, and Dp10 learn successfully (Figure 6b). At 9 months of age, control and Dp10, both female and male, also learn successfully.

#### 3.2.5. Acoustic Startle and Prepulse Inhibition

**Figure 7:**
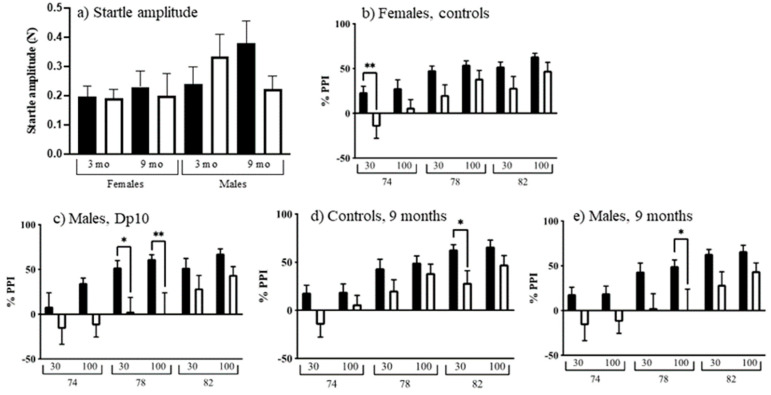
Startle response and prepulse inhibition. (**a**) Startle amplitude. Black bars, control; white bars, Dp10. (**b**–**e**) 30 or 100 ms at 74, 78, or 82 dB. (**b**) Control females, black bars, 3-month-old; white bars, 9-month-old. (**c**) Dp10 males, black bars, 3-month-old; white bars, 9-month-old. (**d**) 9-month-old controls, black bars, male; white bars, female. (**e**) 9-month-old males, black bars, controls; white bars, Dp10. *, *p* ≤ 0.05; **, *p* ≤ 0.01.

Acoustic Startle and prepulse inhibition (PPI) measure cerebellar learning that does not involve motor coordination by assessing sensorimotor gating. As shown in Figure 7a, on startle-only trials (no prepulse), female mice startled less than males (F(1, 102) = 4.676, *p* = 0.0329). Comparisons between the eight age, genotype, and sex groups revealed many differences in PPI. PPI decreased with age in female controls (Figure 7b; F(1, 26) = 5.769, *p* = 0.0238), and also in male Dp10 (Figure 7c; F(1, 26) = 7.018, *p* = 0.0135). Nine-month-old control males showed greater PPI compared to nine-month-old control females (Figure 7d; F(1, 25) = 4.297, *p* = 0.0486). Nine-month-old control males also showed greater PPI compared to nine-month-old Dp10 males (Figure 7e; F(1, 26) = 6.843, *p* = 0.0146). 

## 4. Discussion

The diversity of tasks assessed here illustrates the complexity inherent in modeling DS learning/memory phenotypes in mice. There were no differences among the eight groups of mice in the distance traveled in the open field, nor in behavior in the elevated zero maze, or the Y-maze. In addition, when the performance of individual mice in individual tasks was compared, poor performance in one task did not predict poor performance in another task, e.g., a mouse who managed only a brief time on the rotarod might still have a very fast time in the double-H maze (Supplementary Table S1). Thus, features such as curiosity in a novel environment and basic motor skills were not limiting factors in the performance of more demanding cognitive tasks.

In addition, the performance of male control mice here is consistent in several features to those reported in an aging study with the same inbred strain, C57BL/6J. Shoji et al. (2016) analyzed the behavior of >1700 wild-type male C57BL/6J obtained as control mice from >170 strains of genetically modified mice [74]. They grouped mice in four ages: 2–3, 4–5, 6–7, and 8–12 months, partially overlapping with ages studied here. Similar to our results, Shoji et al. (2016) found an increase in body weight, a decrease in rotarod performance, no change in distance in the open field, and a decrease in the time in the light (relevant to the increase in border time seen here in the open field) between mice 4–5 months and mice 8–12 months of age. This consistency adds support to the data obtained here.

**Figure 8:**
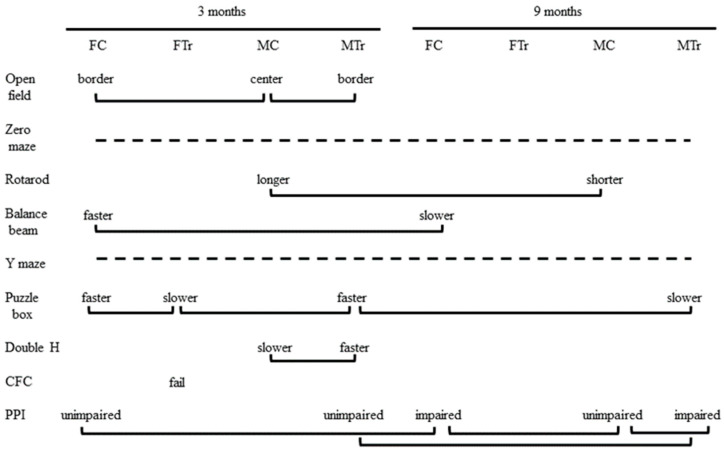
Summary of sex, genotype, and age differences for all tasks. Solid lines indicate comparisons with significant differences between groups. Dashed lines indicate tasks showing no differences between any groups. PPI, prepulse inhibition of acoustic startle. CFC, context fear conditioning.

Figure 8 summarizes the results from all assays. From the nine tasks, there were 13 comparisons significantly affected by genotype, sex, or age. There were five direct effects of genotype that were also sex-specific: female Dp10 at 3 months were impaired in the puzzle box and in CFC, while male Dp10 differed from male controls in the open field at 3 months, and, interestingly, outperformed controls in the double-H maze at 3 months, and were impaired in PPI at 9 months. Relative to sex-matched controls, trisomy also had some protective consequences: unlike female controls, female Dp10 did not lose speed on the balance beam with age and appeared to be protected from an age-associated impairment in prepulse inhibition seen in female controls; and unlike male controls, male Dp10 did not lose ability on the rotarod with age. However, age negatively affected male Dp10, as manifested by relatively impaired performance in the puzzle box and prepulse inhibition at 9 months.

Male Dp10 mice were reported to be unimpaired in CFC at 2–4 months of age [31]; however, subtle impairments were subsequently uncovered with the use of a milder training protocol [73]. This was sex-specific because female Dp10 at 3 months did not show this deficit. In the current work, however, using the same milder protocol, different results were obtained: male Dp10 mice were unimpaired while female Dp10 mice were impaired. This is an unfortunate complication precluding simple interpretation. Notably, mice here were older (although training started at 3 months of age, by the time mice were exposed to CFC, they were closer to 6 months of age) and importantly had been exposed to a variety of experiences, some quite stressful, e.g., multiple novel environments, including the puzzle box where escape from a lighted compartment was the challenge, water in the double-H maze, etc. Possibly female Dp10 were more stressed and less able to learn by the time CFC came around. On the other hand, female Dp10 in the 9-month group (i.e., close to 12 months of age) were successful in learning in CFC.

Male Dp10 mice have also been shown to be impaired in the alternating T-maze [58] at 3, 6, and 9 months of age, but here showed no deficits in the alternating Y-maze. Differences in protocols are again an important consideration. Here, continuous free running in the Y-maze was assessed, while in [58], the T-maze test involved repeated single trials, demanding greater recognition of prior choices and decision making [75].

The puzzle box assesses short- and long-term memory and problem-solving skills and has not previously been used in the evaluation of any DS mouse model. Deficits in puzzle box performance have, however, been shown to arise from lesions in the hippocampus or the prefrontal cortex and from a number of chemical and genetic manipulations, including injection of the NMDA inhibitor, MK-801, alterations in dopamine signaling in the striatum, and knockout of the GluA1 receptor. Each of these manipulations resulted in a specific pattern of trial-dependent deficits [67], with the most severe deficits arising from lesions in the hippocampus that caused significant impairments in every trial. In contrast, here, the Dp10 impairments were largely confined to Trial 5 and most closely resemble the pattern seen in mice with prefrontal cortex lesions. Both female and male Dp10 showed abnormalities in puzzle box performance at 3 months of age; however, while females were impaired, taking significantly longer to solve the escape task in trial 5, males showed enhanced performance, solving the problem more quickly than females. These data suggest sex and age-dependent changes in trisomy in prefrontal cortex function.

The hippocampus has been studied extensively in DS and in DS mouse models, with structural abnormalities and impairment in spatial learning tasks requiring a functional hippocampus well established. The striatum is also involved in spatial learning, but in contrast to the hippocampus, which requires external cues, striatal tasks are solved using egocentric, body-centered, or response learning. The striatal function has recently been shown to be a relative strength in DS, and it has been proposed that the impairment of hippocampal function has led to enhanced striatal abilities [17,18]. Consistent with this concept, here, male Dp10 at 3 months of age performed better than age-matched male controls in the version of the double-H maze requiring an egocentric strategy. This enhancement was not seen in female Dp10, nor in males at 9 months of age.

Abnormalities in cerebellar size and cellular structure have been characterized in DS, and it is noteworthy that it was observations in a mouse model of DS that motivated the initial investigations, and discovery, in people with DS. The cerebellar function is most commonly assessed by rotarod, grip strength, gait analysis, i.e., assays of motor function. Cerebellum, however, is also involved in cognition, executive function, spatial learning, and language processing [76,77,78,79,80]. Prepulse inhibition of the acoustic startle response depends in part on the cerebellum. Female Dp10 appeared to be protected from a sex-dependent decline in PPI seen in female controls, while, conversely, male Dp10 declined with age.

Few comparisons of results here can be made with learning/memory deficits seen in other mouse models of DS for two reasons: (i) no other models have been assessed for performance in the puzzle box, double-H maze, or acoustic startle/prepulse inhibition, and (ii) female mice have been assessed in only a single DS model, the recent TcMAC21 [33], and only in the Morris Water Maze. CFC is a more common task. Both the Ts65Dn and the triple trisomic model were impaired (although the protocols differed from that here) [30,37]. Given that both female and male Dp10 have shown deficits in CFC, depending upon age, experience, and genetic background [73], there is no simple extrapolation between gene content and learning/memory impairment.

The 39 protein-coding genes mapping within the Mmu10 region orthologous to Hsa21 are associated with a diverse array of functions. Not enough is known about them to allow the prediction of candidates for the abnormalities characterized here in trisomy. Previous protein expression analysis of cortex, hippocampus, and cerebellum of female and male wild-type C57BL/6J mice revealed significant sex differences at 8 months of age [53]. These differences were particularly common in the hippocampus, where 40 of the ~100 proteins/protein modifications measured were significantly higher in females vs. males. The sex differences included components of the MTOR and MAPK pathways. Analysis of littermate Dp10 revealed complex patterns of perturbations that were both sex and brain region-specific. Female Dp10, for example, showed many more abnormalities in the cerebellum, while male protein levels were more perturbed in the hippocampus. Such molecular-level sex differences and others to be discovered, e.g., in the striatum, may contribute to, or reflect, the sex-specific abnormalities in tasks such as CFC, the puzzle box, the double-H maze, and/or PPI.

## 5. Conclusions

The age and sex-dependent learning/memory strengths and weaknesses described here for the Dp10 suggest that trisomy of these orthologous Hsa21 genes contributes to the phenotypic landscape of ID in DS. How these trisomic features would influence and be influenced by the effects of genes on Mmu16 and Mmu17 in complete trisomy remains to be determined. The focus in much DS research and in most DS mouse model research has been on the hippocampus and hippocampus relevant tasks, such as the Morris Water (and other) Mazes, CFC, and Novel Object Recognition. While this unified focus aids with consistent interpretations between experiments and simplifies predictions, it may be masking the true complexity of ID in DS. This, in turn, may lead to inaccurate results in the testing of pharmacotherapies. If preclinical evaluations of potential drugs for the rescue of ID in DS do not assay a diverse set of tasks in female and male mice, impairments may go undetected until human clinical trials fail. Certainly, if the trisomy of genes in the Mmu10 orthologous segment continues to be relatively ignored, it will remain difficult to be confident in the validity of mouse models and their results.

## Figures and Tables

**Table 1:** Numbers and weights of mice in each genotype/sex cohort.

		Female						Male					
Age	Range	C	Wt	Range	Tr	Wt	Range	C	Wt	Range	Tr	Wt	Range
3 mo	2.3–3.5 mo	15	27.8 ± 1.3 g	22.4–36.1	15	25.2 ± 0.6 g	22.7–27.3	13	31.4 ± 1.2 g	24.2–41.2	14	27.6 ± 1.6 g	20.5–37.6
9 mo	7.8–10.2 mo	13	33.4 ± 1.7 g	23.1–46.0	13	29.2 ± 1.4 g	21.0–40.5	14	38.2 ± 1.9 g	27.2–50.1	14	27.8 ± 1.3 g	25.1–40.4

Age/Range, months at the start of testing. C, number of control mice; Tr, number of trisomic mice. Wt, weight in grams, mean ± SEM; range, minimum and maximum weights.

## Data Availability

Data are provided in Supplementary Tables S1 and S2 available online and from the authors.

## Supplementary Materials

The following are available online at https://www.mdpi.com/article/10.3390/genes12111697/s1, Tables S1 and S2: Mouse information and Task comparisons.

## Abbreviations

AD	Alzheimer’s disease
CFC	context fear conditioning
DS	Down syndrome
Dp10	Dp10(1)Yey
EZM	elevated zero maze
Hsa21	human chromosome 21
ID	intellectual disability
MWM	Morris water maze
Mm10	mouse chromosome 10
PPI	prepulse inhibition
rpm	rotations per minute
