# Supplementary material for: Trisomy of Human Chromosome 21 Orthologs Mapping to Mouse Chromosome 10 Cause Age and Sex-Specific Learning Differences: Relevance to Down Syndrome
Source: Genes (Basel). 2021 Oct 26;12(11):1697. doi: 10.3390/genes12111697 (PMC8618694; doi:10.3390/genes12111697)
Supplement: Supplementary file 1 [file genes-12-01697-s001.zip › genes-1429400-supplementary.pdf]

Table S1: Mouse information

				balance beam	open field	rotarod	Puzzle Box		
ID	Sex	Gend	Age	fastest time (s)	cm	avg. fall (s)	Trial 2 (s)	Trial 5 (s)	Trial 8 (s)
N2999	F	C	9.00	5.00	7616.23	72.00	5.00	108.00	18.00
S3308	F	C	9.00	14.00	3021.52	279.67	18.00	55.00	17.00
S3310	F	C	9.00	9.00	3652.47	153.33	5.00	31.00	9.00
S3319	F	C	9.00	fell 3 times	3127.05	132.67	94.00	52.00	29.00
S4434	F	C	9.00	10.00	3839.01	171.00	21.00	82.00	41.00
S4435	F	C	9.00	no data	3530.34	159.67	11.00	78.00	46.00
S4520	F	C	9.00	fell 3 times	4323.66	184.33	7.00	105.00	21.00
S4522	F	C	9.00	14.00	4781.67	160.33	7.00	152.00	49.00
S4523	F	C	9.00	9.00	4432.68	152.33	12.00	108.00	111.00
S4526	F	C	9.00	fell 3 times	5632.50	193.33	9.00	169.00	42.00
S5053	F	C	9.00	no data	3848.64	167.33	5.00	70.00	20.00
S5054	F	C	9.00	no data	5487.56	207.00	4.00	91.00	30.00
S9664	F	C	9.00	no data	4076.72	199.67	9.00	96.00	20.00
									34.85
S1994	F	C	3.00	5.00	4537.74	284.33	22.00	30.00	31.00
S1999	F	C	3.00	6.00	4034.13	123.67	7.00	52.00	14.00
S2494	F	C	3.00	5.00	3737.47	229.67	19.00	104.00	15.00
S2512	F	C	3.00	6.00	3937.97	277.00	8.00	72.00	13.00
S2513	F	C	3.00	5.00	4103.63	167.00	10.00	113.00	22.00
S3678	F	C	3.00	7.00	4281.93	236.67	8.00	52.00	14.00
S3679	F	C	3.00	6.00	4535.92	190.67	10.00	48.00	17.00
S4204	F	C	3.00	7.00	4567.51	178.67	13.00	87.00	33.00
S4207	F	C	3.00	3.00	4709.95	125.00	9.00	92.00	33.00
S5127	F	C	3.00	no data	6052.81	283.00	13.00	217.00	24.00
S5163	F	C	3.00	no data	3754.79	191.00	10.00	48.00	24.00
S9073	F	C	3.00	4.00	4327.34	261.33	11.00	33.00	39.00
S9075	F	C	3.00	3.00	5000.18	138.33	18.00	76.00	13.00
S9079	F	C	3.00	6.00	5260.72	76.00	14.00	59.00	22.00
S9081	F	C	3.00	5.00	no data	175.33	16.00	99.00	27.00
S0904	F	Ts	9.00	10.00	6090.75	247.00	8.00	50.00	16.00
S0905	F	Ts	9.00	7.00	5654.58	261.00	7.00	168.00	14.00
S0906	F	Ts	9.00	5.00	6293.72	262.50	11.00	20.00	27.00
S0907	F	Ts	9.00	6.00	5164.79	240.00	17.00	153.00	25.00
S1687	F	Ts	9.00	fell 3 times	4396.04	140.67	10.00	155.00	14.00
S3307	F	Ts	9.00	14.00	4382.26	274.67	18.00	33.00	25.00
S3309	F	Ts	9.00	13.00	3895.14	280.33	17.00	59.00	65.00
S3311	F	Ts	9.00	17.00	5351.64	109.67	5.00	31.00	30.00
S3313	F	Ts	9.00	fell 3 times	4196.58	190.33	17.00	82.00	19.00
S3314	F	Ts	9.00	12.00	4147.78	122.67	16.00	78.00	36.00
S3315	F	Ts	9.00	no data	4385.10	124.33	10.00	160.00	38.00
S5052	F	Ts	9.00	no data	4708.87	197.00	3.00	68.00	29.00
S5055	F	Ts	9.00	no data	4846.08	237.67	7.00	54.00	15.00
S1995	F	Ts	3.00	10.00	4213.73	300.00	11.00	75.00	22.00

S1997	F	Ts	3.00	4.00	4227.43	102.33	16.00	150.00	22.00
S2100	F	Ts	3.00	17.00	3970.37	94.97	35.00	110.00	11.00
S2493	F	Ts	3.00	4.00	5829.73	129.33	7.00	255.00	11.00
S2495	F	Ts	3.00	4.00	4329.30	201.33	13.00	54.00	15.00
S2511	F	Ts	3.00	6.00	4465.34	187.00	7.00	76.00	20.00
S3677	F	Ts	3.00	5.00	5592.57	228.00	8.00	136.00	19.00
S3680	F	Ts	3.00	14.00	5001.49	239.33	6.00	104.00	21.00
S4203	F	Ts	3.00	3.00	3266.28	268.00	35.00	115.00	35.00
S4205	F	Ts	3.00	4.00	4900.55	249.00	13.00	43.00	22.00
S4206	F	Ts	3.00	6.00	5710.86	166.67	17.00	62.00	47.00
S5128	F	Ts	3.00	no data	5174.23	188.67	13.00	117.00	25.00
S5164	F	Ts	3.00	no data	4459.04	254.67	10.00	87.00	19.00
S9074	F	Ts	3.00	5.00	4626.03	150.33	5.00	84.00	31.00
S9080	F	Ts	3.00	4.00	4759.14	50.00	9.00	105.00	94.00
N2975	M	C	9.00	no data	5942.74	76.00	17.00	74.00	72.00
N2976	M	C	9.00	6.00	5132.72	111.33	22.00	134.00	33.00
N2978	M	C	9.00	16.00	6987.10	127.67	12.00	170.00	110.00
S0901	M	C	9.00	9.00	5289.08	129.33	16.00	62.00	25.00
S3304	M	C	9.00	no data	3638.90	220.33	46.00	84.00	38.00
S3305	M	C	9.00	10.00	4079.15	212.33	22.00	112.00	8.00
S3306	M	C	9.00	10.00	5141.78	196.33	14.00	108.00	67.00
S3312	M	C	9.00	fell 3 times	4476.75	61.00	18.00	43.00	32.00
S3317	M	C	9.00	fell 3 times	4610.51	89.67	18.00	70.00	84.00
S4432	M	C	9.00	7.00	5536.42	114.67	13.00	254.00	115.00
S4521	M	C	9.00	6.00	4605.07	228.67	26.00	60.00	32.00
S9651	M	C	9.00	no data	6119.00	73.67	6.00	148.00	28.00
S9660	M	C	9.00	no data	5303.34	105.67	11.00	57.00	17.00
S9661	M	C	9.00	no data	5867.17	123.00	8.00	93.00	28.00
437.00	M	C	3.00	9.00	4618.54	193.67	5.00	53.00	85.00
439.00	M	C	3.00	7.00	5359.40	172.00	10.00	65.00	41.00
S2492	M	C	3.00	4.00	4721.10	227.33	28.00	59.00	31.00
S3673	M	C	3.00	15.00	3161.57	241.67	23.00	131.00	26.00
S3674	M	C	3.00	4.00	4075.48	190.33	11.00	52.00	25.00
S3675	M	C	3.00	5.00	4022.13	161.67	13.00	86.00	23.00
S5124	M	C	3.00	no data	4994.96	167.33	13.00	132.00	37.00
S5125	M	C	3.00	no data	5558.30	147.67	9.00	243.00	146.00
S5126	M	C	3.00	no data	5389.88	216.00	24.00	94.00	63.00
S5160	M	C	3.00	no data	5307.12	266.33	4.00	60.00	22.00
S9065	M	C	3.00	5.00	4397.80	288.00	14.00	38.00	38.00
S9066	M	C	3.00	6.00	4058.09	186.67	7.00	96.00	33.00
S9067	M	C	3.00	6.00	no data	259.00	13.00	75.00	55.00
N2979	M	Ts	9.00	6.00	6364.74	263.67	13.00	117.00	25.00
N2998	M	Ts	9.00	no data	3996.19	104.00	13.00	71.00	32.00
S0902	M	Ts	9.00	6.00	5909.31	182.67	4.00	95.00	19.00
S1686	M	Ts	9.00	10.00	5853.30	183.67	15.00	36.00	10.00
S3316	M	Ts	9.00	fell 3 times	4447.63	252.00	12.00	50.00	50.00

S4433	M	Ts	9.00	13.00	4028.37	143.33	13.00	52.00	30.00
S4517	M	Ts	9.00	fell 3 times	4222.50	150.00	10.00	124.00	79.00
S4518	M	Ts	9.00	5.00	5078.19	226.00	22.00	184.00	38.00
S9554	M	Ts	9.00	no data	4835.79	127.00	10.00	103.00	46.00
S9555	M	Ts	9.00	no data	4878.84	262.00	7.00	300.00	32.00
S9556	M	Ts	9.00	no data	4169.22	154.00	6.00	89.00	28.00
S9557	M	Ts	9.00	no data	4108.82	285.33	8.00	44.00	17.00
S9652	M	Ts	9.00	no data	5198.55	84.33	10.00	127.00	22.00
S9659	M	Ts	9.00	no data	3869.15	214.00	16.00	112.00	32.00
438.00	M	Ts	3.00	6.00	5165.06	174.00	13.00	49.00	39.00
S1990	M	Ts	3.00	7.00	2937.45	172.00	14.00	86.00	38.00
S1991	M	Ts	3.00	4.00	3498.85	140.33	7.00	53.00	26.00
S1992	M	Ts	3.00	6.00	3270.94	226.67	15.00	64.00	34.00
S1993	M	Ts	3.00	6.00	4123.63	156.33	7.00	69.00	30.00
S1998	M	Ts	3.00	5.00	3790.07	281.33	10.00	58.00	29.00
S2510	M	Ts	3.00	6.00	3802.10	219.67	10.00	30.00	36.00
S3676	M	Ts	3.00	6.00	4233.29	235.33	17.00	65.00	28.00
S4202	M	Ts	3.00	7.00	3636.41	204.00	8.00	73.00	27.00
S5123	M	Ts	3.00	no data	4145.92	226.00	20.00	84.00	30.00
S5159	M	Ts	3.00	no data	5771.62	115.67	8.00	46.00	28.00
S5161	M	Ts	3.00	no data	4848.18	230.33	16.00	57.00	28.00
S5162	M	Ts	3.00	no data	5138.35	235.00	18.00	49.00	50.00
S9064	M	Ts	3.00	8.00	4187.14	300.00	15.00	84.00	123.00

Table S2: Task comparisons

Double-H	
Avg. Day 4 (s)	
5.75	0
13.25	0
27.25	
42.75	
8.00	
9.75	
14.25	
32.00	
28.75	
32.25	
5.75	
24.00	
57.00	
9.75	
17.00	
51.75	
28.00	
42.75	
27.25	
25.75	
29.75	
20.75	
17.25	
17.25	
12.25	
15.25	
28.75	
13.25	
19.75	
22.50	
24.00	
17.75	
7.75	
14.75	
31.00	
10.50	
6.00	
21.50	
27.75	
30.75	
9.25	
15.50	

31.50	
20.00	
5.00	
17.00	
43.50	
49.50	
3.25	
11.00	
10.50	
29.75	
32.25	
9.25	
2.25	
19.75	
15.50	
4.25	
20.75	
11.50	
21.25	
10.00	
25.25	
28.75	
11.75	
27.00	
16.25	
8.25	
7.25	
9.00	
33.50	
17.00	
10.50	
6.25	
41.50	
15.25	
7.00	
24.50	
20.25	
20.00	
10.00	
12.00	
9.75	
7.50	
10.00	
9.75	
9.00	
22.25	

6.50	
16.00	
19.00	
2.50	
11.50	
17.25	
15.00	
26.25	
9.00	
11.00	
10.00	
7.75	
13.50	
6.25	
7.25	
17.00	
2.75	
6.00	
16.75	
18.25	
8.50	
17.75	
2.00	
